# Aggregating Different Scales of Attention on Feature Variants for Tomato Leaf Disease Diagnosis from Image Data: A Transformer Driven Study

**DOI:** 10.3390/s23073751

**Published:** 2023-04-05

**Authors:** Shahriar Hossain, Md Tanzim Reza, Amitabha Chakrabarty, Yong Ju Jung

**Affiliations:** 1 Department of Computer Science and Engineering, BRAC University, Dhaka 1212, Bangladesh; 2 School of Computing, Gachon University, Seongnam 13120, Republic of Korea

**Keywords:** transformers, EANet, MaxViT, CCT, PVT, tomato leaf disease, attention

## Abstract

Tomato leaf diseases can incur significant financial damage by having adverse impacts on crops and, consequently, they are a major concern for tomato growers all over the world. The diseases may come in a variety of forms, caused by environmental stress and various pathogens. An automated approach to detect leaf disease from images would assist farmers to take effective control measures quickly and affordably. Therefore, the proposed study aims to analyze the effects of transformer-based approaches that aggregate different scales of attention on variants of features for the classification of tomato leaf diseases from image data. Four state-of-the-art transformer-based models, namely, External Attention Transformer (EANet), Multi-Axis Vision Transformer (MaxViT), Compact Convolutional Transformers (CCT), and Pyramid Vision Transformer (PVT), are trained and tested on a multiclass tomato disease dataset. The result analysis showcases that MaxViT comfortably outperforms the other three transformer models with 97% overall accuracy, as opposed to the 89% accuracy achieved by EANet, 91% by CCT, and 93% by PVT. MaxViT also achieves a smoother learning curve compared to the other transformers. Afterwards, we further verified the legitimacy of the results on another relatively smaller dataset. Overall, the exhaustive empirical analysis presented in the paper proves that the MaxViT architecture is the most effective transformer model to classify tomato leaf disease, providing the availability of powerful hardware to incorporate the model.

## 1. Introduction

Countries that bank on the cultivation of crops for survival tend to grow mostly green crops and vegetables. Tomato cultivation is popular among these crops and is grown worldwide. However, tomatoes and their leaves are often plagued by a variety of diseases mostly due to fungus attacks and adversary weather conditions. In either case, it is imperative to treat the disease but more crucial to find the type of disease a particular leaf is infected with. With different classes of leaf diseases, it became increasingly more difficult to correctly classify them with traditional methods. Computer Vision and Deep Learning (DL) techniques have been automating the analysis of different categories of data for some time. DL-based classification techniques make the disease classification process faster and more accurate.

Recently, huge amounts of attention have been given to the field of image classification through the introduction of Vision Transformers (ViT) which originates originally from Natural Language Processing (NLP). Transformer-based models are more robust and recent studies have confirmed that they produce better results compared to the DL-based models. Many applications are taking the aid of transformers-based models. For example, M. Kaselimi et al. [1] employed a ViT model to demonstrate multilabel classification of satellite pictures in deforestation, whereas the previous studies in [2,3] used ViT to study remotely sensed images. T. Wang et al. [4] presented a noble ViT model where they suggested a suitable partition method to reduce the impact of data localization in the image and boost the efficacy of computation and memory access. In [5], a review of ViT models has been demonstrated, along with their applications, benefits, and drawbacks.

Transformer-based models employ an attention-based approach that offers context for any location in the input sequence. ViT-based models separate the images into visual tokens, in contrast to convolutional-based models a series of convolution and pooling layers are utilized to extract features from images. The transformer-based models may alter an image by breaking it up into fixed-size patches, precisely embedding each one, and providing positional embedding as an input to the model encoder. However, ViTs are data hoarders [6]; therefore, a huge amount of data must be fed to the model in order for it to deliver a good outcome. The self-attention layer of ViT lacks location inductive bias (LIB). ViTs need more information as a result.

All the existing studies have used CNN-based models [7,8,9] for the tomato leaf disease classification task. However, in this study, we performed intensive experiments on recent transformer-based models, which thrive based on the aggregation of different attention and feature types. We want to explore these models due to the transformers having different attributes that aid in the classification task. In this paper, we explore the different state-of-the-art ViT models by implementing these on a large dataset consisting of numerous classes of tomato leaf disease. Furthermore, we verified our findings on a second smaller dataset. We aim to use these models in the future for other classification works such as bark texture classification [10]. Contributions of this paper are as follows:

- Four state-of-the-art(SOTA) Vision Transformer models named CCT, PVT, EANet, and MaxViT have been implemented and their respective parameters alongside produced outputs are compared.
- Various metrics are utilized to assess the efficacy of the models including accuracy curves, loss curves, confusion matrices, receiver operating characteristic curves, and classification reports.
- To the best of our knowledge, this is the first paper that has done comprehensive research work on tomato leaf diseases using Vision Transformer architectures.

The paper is organized as follows: Section 2 covers related research on the vision transformer domain and its use in several medical domains. We describe the architecture of our proposed model in Section 3 and compare it to the comparator models. The results of each model are displayed in Section 4 and Section 5 in terms of accuracy, confusion matrices, and classification reports. Our conclusion is drawn in Section 6.

## 2. Related Works

This section overviews a summary of the SOTA literature on the subject of leaf diseases, with a focus on the classification of tomato leaf diseases utilizing DL methods.

The literature utilizes either machine learning-based or DL-based methods for the detection and classification of tomato leaf diseases. Several classification models, such as AlexNet, VGG16, and VGG19, have been used in various studies such as [7,8]. Authors have used AlexNet [11] as their DL model, VGG16 [12], and VGG19 [13] as neural network models for their detection and for classification, they have used the KNN [14] model. They have used the PlantVillage dataset in their study. The effect of hyperparameter tuning and dataset size on the models’ performance has also been studied, indicating that an increase in the number of images improves overall performance. A summary paper [15] shows an extensive study on the state-of-the-art work on leaf disease classification. Additionally, modified methods have been proposed such as in [16], a 14-layer CNN model was developed for identifying plant diseases. In their proposal, they utilized different image augmentation techniques to increase the number of images and balance the class. The authors have run their model for 1000 epochs in a multi-graphics processing environment. Their model gave over 99% overall classification accuracy. A modified faster RCNN model was proposed in [17] for detecting and locating tomato leaf diseases. For feature extraction, the authors have used ResNet in place of VGG16, and the proposed model gave around 2.7% better accuracy compared to the regular faster RCNN model. A robust approach has been presented for tomato plant leaf disease localization and classification in [18]. In this paper, the authors have proposed a method based on the faster RCNN. Overall, these studies demonstrate the potential of DL techniques for the accurate and efficient classification of tomato leaf diseases.

A CornerNet model based on DenseNet-77- has been used in [19] for the localization and classification of tomato plant leaf diseases. In this proposed method, DenseNet-77 is used for generating the feature vector which is used to detect and localize the tomato leaf disease using the single-stage CornerNet model. Improved YOLO V3-based method has been used in [20] for tomato leaf disease detection and classification. Since the disease spot is tiny compared to the plant size, the YOLO V3 method has been modified to get better accuracy with a faster processing time. The proposed model improved the YOLO V3 using image pyramid [21] through the utilization of multi-scale feature detection, object bounding box dimension clustering [22], and multi-scale training. The accuracy result of this improved method shows improvement in comparison to other methods in the literature along with fast detection time. A DL-based tomato disease classification and symptoms visualization method has been presented in [9]. This method used more than 14,000 tomato leaf images to classify 9 different diseases using CNN models. Their study shows that DL models give better classification accuracy than compared to shallow methods such as SVM, and Random Forest. A lightweight CNN model has been proposed in [23] in comparison to the traditional heavyweight CNN methods. The proposed method has used only 8 hidden layers to detect 10 different tomato leaf diseases with higher accuracy than compared to other available methods. In [24], an attention-based method has been developed to detect tomato leaf disease. This method used over 15,000 images having 10 classes, 9 diseased, and 1 healthy. As the backbone of the model in the proposed model, resNet was used where the work achieves a higher accuracy with fast computation. Table 1 shows a summary of the tomato leaf disease detection works presented in the literature. In [25], CNN is used for the early detection of tomato leaf disease. A dataset of size 300 is used for the experiment which has been done on a google colab environment. Various colors, textures, and edges of the input images are considered in the analysis. The authors have achieved an accuracy of more than 98% in this work.

Table 1 shows a summary of some of the research conducted on identifying plant leaf diseases. It shows that none of the reported methods have used the more advanced, accurate, and SOTA ViT-based models. In the remaining section of this paper, the use of ViT-based models in identifying tomato leaf diseases is highlighted and a comparison of accuracies alongside other related parameters is given.

## 3. Proposed Methodology

This section explains the methodology used in the study, from data collection to visual representations, to demonstrating the contributions made in the paper.

### 3.1. Data Collection

**Figure 1:**
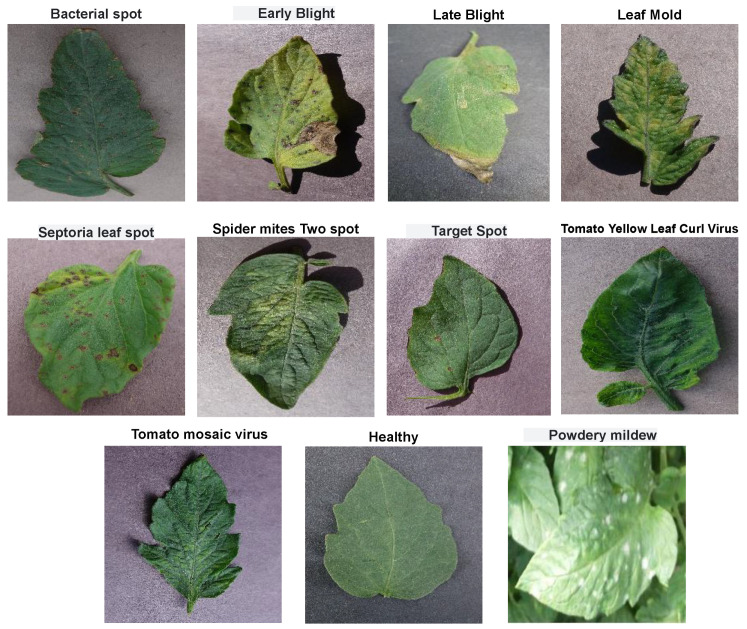
Sample of the first dataset used in the study.

We collected the dataset from kaggle [29]. This dataset comprises a total of 20,000 images with 11 different classes having 10 classes consisting of images with 10 different types of diseases infesting tomato leaves and 1 class having healthy leaves. A workstation with Ryzen 5950X, 64 GB RAM, and NVIDIA GeForce RTX 3090 is used to implement all the models. Figure 1 shows sample images of each of 11, different classes of tomato leaf images, while Table 2 shows the class distribution of the classes in terms of training and validation directories.

**Figure 2:**
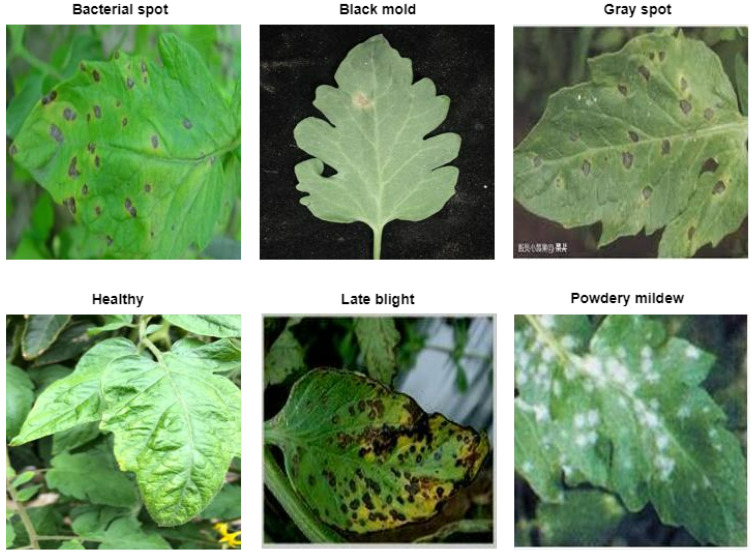
Sample of the second dataset used in the study.

Additionally, we utilized the plantvillage dataset [30] to further verify the findings from the first dataset. This second dataset consists of 6 classes and a total of 4972 images after augmentation. The class information and samples from the classes are visualized in Figure 2. Besides, the training and validation split of the dataset is given in Table 3.

### 3.2. Model Seclection

In this research, we implement four ViT-based models to identify different types of tomato leaf diseases. Most of these transformers combine multiple types of features or utilize various types of attention mechanics. Their mechanisms are discussed below.

#### 3.2.1. EANet

**Figure 3:**
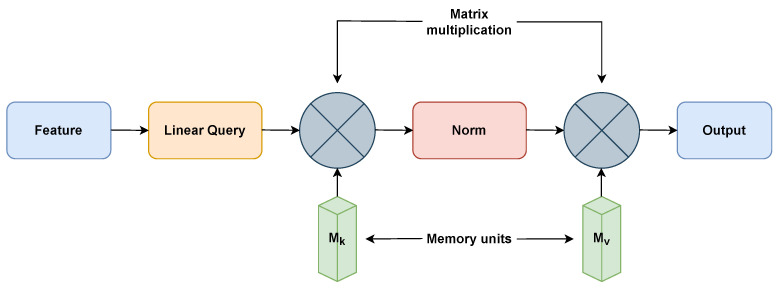
External-attention for EANet model [31].

EANet [31] uses the external attention mechanism and is built on two external, small, teachable, and shared memories, $M_{k}$, and $M_{v}$, as shown in Figure 3. In order to boost performance and computational efficiency, EANet is used to remove patches with redundant and unnecessary information. Two cascaded linear layers and two normalization layers are used to implement the external attention. The attention between input pixels and an external memory unit is determined by the EANet algorithm using the following Equations (1) and (2), where *F* is the input feature. Last, the attention in *A* produces a newer version of $M_{v}$’s input features.

The external attention mechanism of EANet separates it from the regular ViTs. The external attention knowledge may come from incorporating additional images, textual descriptions, or pre-trained models into the image classification process. EANet incorporates multi-head attention to process these input images and external knowledge simultaneously. Afterward, the features from the two sources are combined via semantic fusion. Regular ViTs frequently require a lot of training data. However, EANet excels in specific situations where there is a limitation of training data.

(1) $$A=Norm(FM_{k}^{T}).$$

(2) $$Fout=AM_{v}.$$

#### 3.2.2. MaxViT

**Figure 4:**
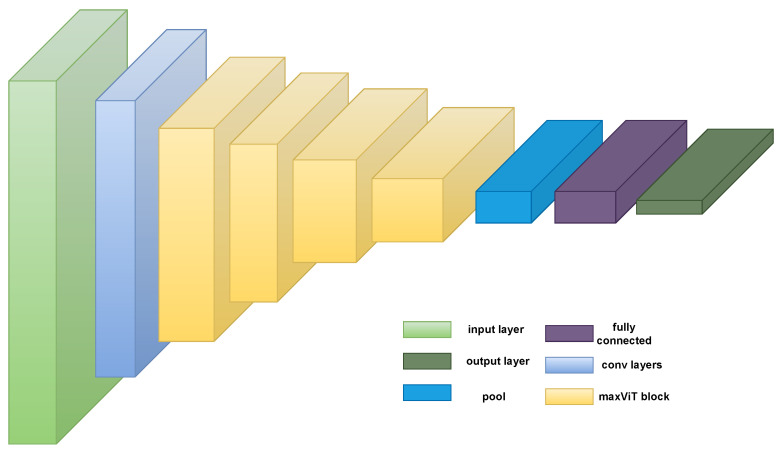
MaxViT architecture [32]. Note that this model uses both local and global mechanisms via the maxViT block.

Multi-axis attention [32], a model of effective and scalable attention that has two components—blocked local and dilated global attention—is introduced by the authors. These design decisions enable linearly complicated global-local spatial interactions at any input resolution. The authors also introduce a new architectural component by successfully fusing their proposed attention model with convolutions, and they suggest a straightforward hierarchical vision backbone by simply repeating the fundamental building block throughout many phases. The MaxViT architecture is shown in Figure 4.

Generally, ViTs are designed to divide images into patches and input them in a transformer model to learn the global representation of the image patches. Meanwhile, the MaxViT model can capture both local and global interactions between image patches due to its multi-axis attention. Additionally, unlike normal ViTs, MaxVit divides the input image into patches of various sizes using a hierarchical patch partitioning approach, which enables the model to capture both fine- and coarse-grained characteristics in the input data. The size of the patches is adjusted dynamically based on the intricacy of the input images. Additionally, MaxViT is designed to be highly scalable, enabling it to be applied to very large images.

**Figure 5:**
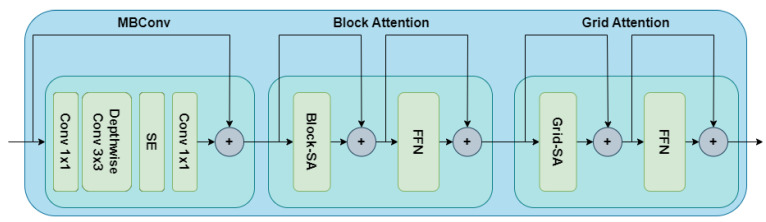
MaxViT block to capture both local and global attention information [32].

Figure 5 showcases the highlight of a basic MaxViT block. The block attention module helps to capture the local attention information. Meanwhile, the grid attention module helps to capture the global attention information. An MBConv block is also added at the beginning since the presence of this layer helps to improve performance.

#### 3.2.3. CCT

**Figure 6:**
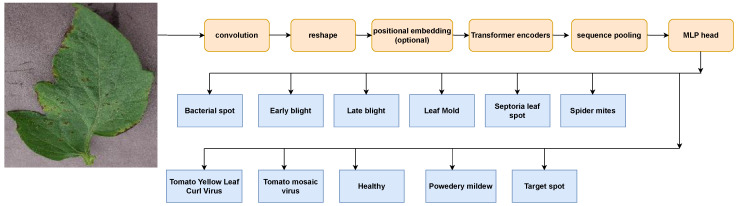
Compact Convolutional Transformer (CCT) used as classifier.

To optimize the display benefits of convolution and transformer, convolution and transformer are combined into CCT [33]. If local information is used effectively, a convolutional approach is used instead of the non-overlapping patches used in CCT’s standard vision transformer. A technique called probabilistic depth tuning, which is similar to dropout, but in which a series of slices is arbitrarily discarded at stochastic depth, is used to prevent the vanishing gradient problem in CCT. In CCT, data are sent through a transformer encoder and sequenced after convolutional tokenization. The possibility of multiple types of morphologies of tomato leaf diseases is provided by sequence pooling MLP headers. Figure 6 unfolds the entire CCT operation.

The combination of transformer blocks and convolutional layers set CCT apart from regular ViT, which generally consists of transformer blocks only. The convolutional layers and the transformer blocks are connected through skip connections, allowing the model to learn a more efficient representation of the input image by combining the features of both convolutional and transformer architectures. CCTs are also a rather efficient extension of the regular ViTs since it uses a variant of the transformer block called the ‘Compressed Transformer’ that reduces the number of parameters and it incorporates parameter sharing which allows the same set of parameters for all patches in a given layer of the transformer. As a result, CCT can drastically lower memory requirements and computational costs. The components of CCT as shown in the figure are discussed in brief below.

Positional Embedding: To introduce spatial information into the sequence, positional embedding is utilized. As the model is not aware of the spatial association between tokens, incorporating additional information that signifies the same can be beneficial. Generally, this is accomplished by either training an embedding or assigning weights to the tokens from two sine waves having high frequencies, which aids the model in recognizing the positional relationship among the tokens.

Transformer Encoder: A transformer encoder is made up of multiple encoding layers that are stacked one on top of the other. Each encoder layer is composed of two sub-layers, namely Multi-Headed Self-Attention (MHSA) and a Multi-Layer Perceptron (MLP) head. Before each sub-layer, a layer normalization (LN) is added, and after that, there is a residual connection to the next sub-layer.

Classification: In ViTs, an additional class token is often incorporated into the sequence of embedded patches, which denotes the class attribute of an entire image, and its state after the transformer encoder can be utilized for classification. The class token comprises implicit information and, via self-attention, accumulates more details about the sequence, which is subsequently employed for classification.

#### 3.2.4. PVT

The challenges of adapting a Transformer to multiple density prediction jobs are overcome by Pyramid Vision Transformer (PVT) [34]. According to the architects of this architecture, PVT has a number of advantages over earlier works. First of all, In order to achieve a high output resolution the dense partitions of the image can be utilized to train PVT, which is important for dense predictions, in contrast to ViT, which typically has low-resolution outputs and high computational and memory costs. PVT can also use a progressive shrinking pyramid to reduce computations of large feature maps. Secondly, PVT, which may be used to replace CNN backbones in a variety of vision applications without the need for convolutions, inherits the advantages of both CNN and the Transformer. Additionally, by completing comprehensive trials, the researchers validate PVT and demonstrate that it improves the performance of numerous downstream activities.

**Figure 7:**
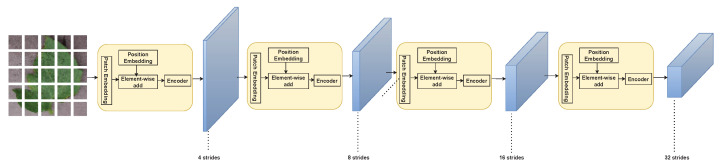
PVT architecture [34].

Figure 7 shows the PVT architecture where there are four stages, each made up of a patch embedding layer and a Bi-layer Transformer encoder, which make up the overall model. The output resolution of the four stages gradually decreases from high (4-stride) to low (32-stride), following a pyramidal shape.

Overall, the key difference between PVT and regular ViT is that PVT incorporates a multi-scale feature aggregation approach that allows it to better handle images with varying scales, while regular ViT uses a fixed-size patch-based approach. The multi-scale feature aggregation approach allows PVT to achieve better results compared to the regular ViTs.

Finally, as we can see, each of the variants of ViT employed in this experiment has a unique attribute that distinguishes it from the regular Vits and helps to improve the overall performance. These ViT variants are also state-of-the-art, outperforming the regular ViTs on large-scale datasets such as ImageNet, Cifer, Coco, etc. Due to this SOTA performance and the distinctive aspects of the ViT variants, we decided to use them in our empirical analysis.

### 3.3. Comparison of Model Parameters

We used NVIDIA GeForce RTX 3090 to run all of the models. To have a fair comparison between the models we ran each of them for 100 epochs. We chose the softmax activation function for the models as this is a multiclass classification task and AdamW is selected as the optimizer.

EANet takes the least amount of time to complete the training compared to the remaining three models. It takes less than half the time to go through 100 epochs in contrast to the rest of the models as stated in Table 4. The reason for EANet having the least amount of time complexity is due to the model having the least number of total parameters. Having a higher number of parameters increase the complexity of the model and makes the model computationally more expensive requiring greater resources. However, having a large number of parameters suggests that it can solve more complicated functions than other models. Additionally, it enables the application of powerful regularization to avoid overfitting.

**Figure 8:**
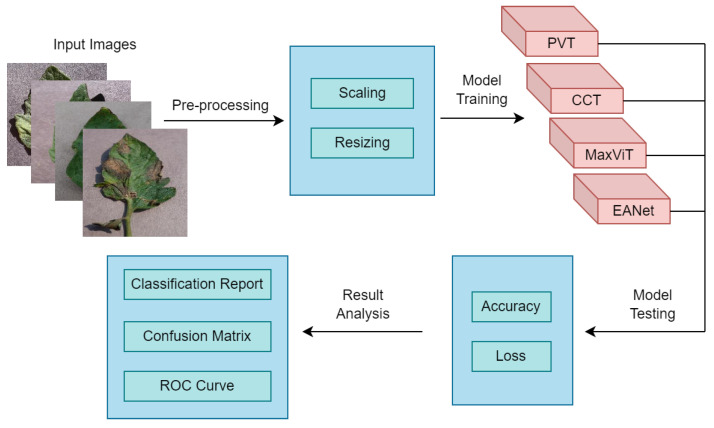
Summary of the methodology.

Overall, the entire methodology can be summarised as given in Figure 8. First, we collected the dataset and performed some pre-processing on it. Afterward, four attention-based transformer models have been deployed and their accuracy alongside loss values have been tested on the test dataset. Finally, their results have been compared in terms of Classification reports, Confusion matrices, and Receiver Operating Characteristic (ROC) curves.

## 4. Performance Evaluation Metrics

In order to evaluate the performance of the implemented models we have used numerous performance evaluation metrics. These include training and loss curves, confusion matrices, classification reports, and ROC curves.

**Figure 9:**
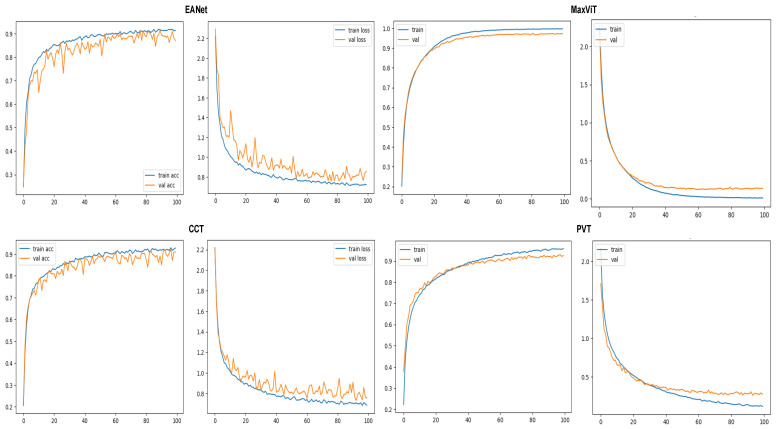
Accuracy and loss curves for the models.

**Figure 10:**
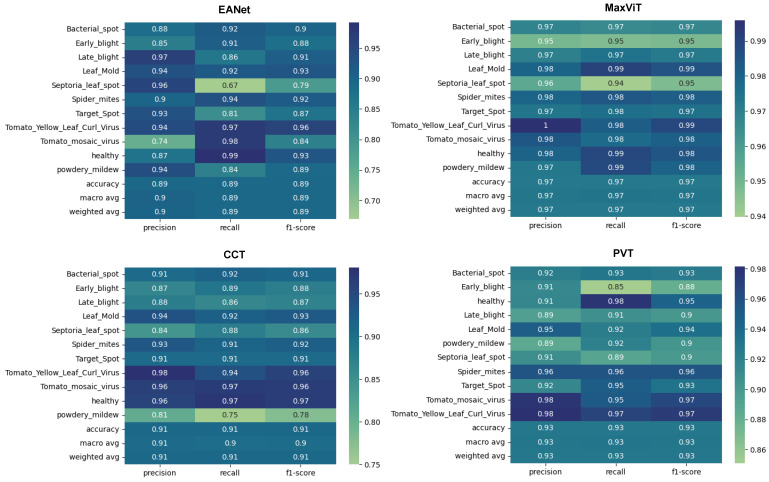
Classification report.

Training and loss curves at Figure 9 show the progress of the models’ classification performance with increasing epochs. Accuracy curves reaching the highest percentage value having a smoother shape with minimum zigzag/fluctuation are considered to produce the best results. The confusion matrix is a two-dimensional array that contrasts the true label with the expected category labels. This helps to keep a track of the number of images that are correctly classified and misclassified by the models. The classification report in Figure 10 unfolds the values of Precision, Recall, and F1 score for the vision transformer models.

Precision (*P*), refers to the proportion of correctly predicted results and the total number of observations, which are positively classified. Precision can be defined as,

(3) $$P=\frac{T_{p}}{T_{p}+F_{p}}.$$

Recall (*R*) is determined as the ratio of correctly predicted results and all evaluations of the original class.

(4) $$R=\frac{T_{p}}{T_{p}+F_{n}}.$$

The *F*1-score (*F*1) calculates a single score by averaging precision and recall.

(5) $$F1=\frac{2\times precision\times recall}{precision+recall}.$$

In the above set of equations,

- $T_{p}$ = True Positive.
- $F_{p}$ = False Positive.
- $F_{n}$ = False Negative.
- $T_{n}$ = True Negative.

Additionally, we used ROC curves, which are performance indicator that works on different threshold levels addressing classification issues. The area under the curve (AUC) stands for the level or measurement of separability, and ROC is a probability curve. It illustrates how much the performance of a particular model may vary between classes.

## 5. Results Analysis

### 5.1. Results on Selected Tomato Leaf Disease Dataset

#### 5.1.1. Accuracy and Loss Curves

The metrics are plotted after 100 epochs of training on each of the selected models. The accuracy and loss curves for the models are provided in Figure 9. The validation accuracy and loss curves of the EANet and CCT models exhibit observable fluctuations after reaching 70% training accuracy, which suggests a potential for more frequent encounters with local maxima. This also likely indicates a slight overfitting issue as these models have a much lower number of parameters compared to the other two models. In contrast, MaxVIT and PVT’s accuracy and loss curves show far fewer spikes, suggesting a smoother learning process thanks to the huge number of parameters that these models have.

The smooth learning curve of PVT and MaxViT also transitions into better accuracy scores. Figure 10 shows that the EANet and CCT models, with accuracy scores of 91% and 89%, respectively, are less accurate than the other variants. Meanwhile, PVT and MaxVIT, who are smooth learners, score at 93% and 97% accuracy, respectively. In terms of pure accuracy, MaxVIT surpasses the other models by a noticeable margin, with a 4% improvement over the next leading model PVT.

#### 5.1.2. Classification Report

Interestingly, different models showcase efficiency over different classes. EANet showcases a noticeable decline in the recall score when classifying Septoria_leaf_spot class, with a recall score of only 67%. It also achieves a relatively low precision score when classifying Tomato_mosaic_virus class, with a precision score of 74%. Maxvit generally performs very well on all the classes apart from getting slightly lower precision, recall, and f1-score for Early_blight and Septoria_leaf_spot classes compared to the other classes. However, even the scores on the lower side of the spectrum stay well around 95%. CCT showcases weaker performance on the powedery_midew class with an 81% precision score, 75% recall score, and 78% f1 score while PVT performs worst for the Early_blight class with a precision, recall, and f1 score of 91%, 85%, and 88%, respectively. As different models showcase performance decline in different classes, perhaps an ensemble of all the models might work better to improve the classification score of EANet, CCT, and PVT. However, it would still probably be fairly difficult to overcome MaxVIT in terms of raw precision, recall, and f1 scores. Meanwhile, in terms of stronger performances, all the models perform really well to detect healthy leaves and the leaves with tomato_yellow_mosaic_virus, with strong results across precision, recall, and f1 scores.

#### 5.1.3. Confusion Matrices

**Figure 11:**
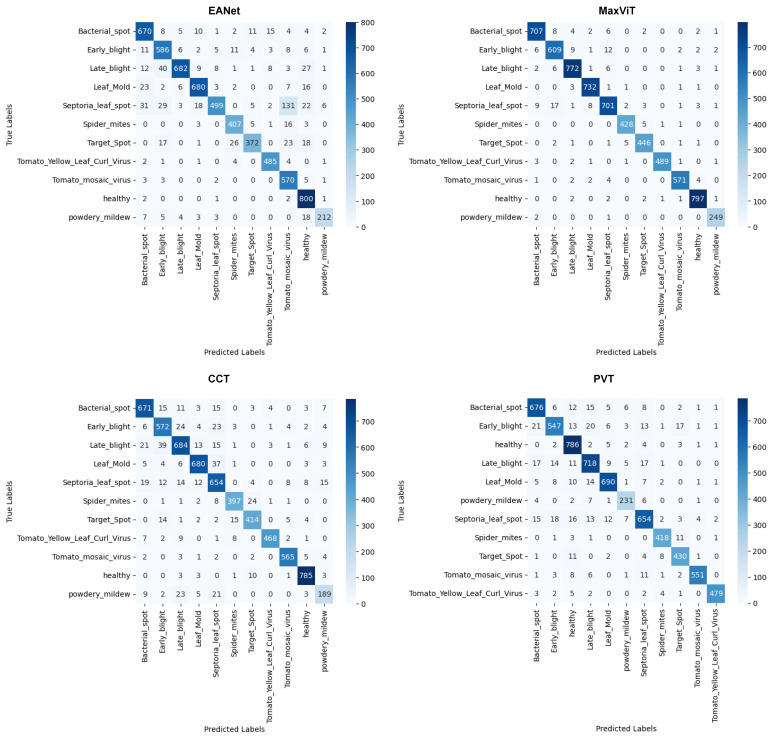
Confusion matrices.

The confusion matrices provided in Figure 11 provide us with a better idea of the misclassified images. Looking at the results produced by EANet, around 131 images of Septoria_leaf_spot are misclassified as Tomato_mosaic_virus. This large amount of misclassification resulted in a low recall and precision scores for the respective classes. Such a massive discrepancy is not present in the results for MaxVit, CCT, and PVT models. However, there are slight misclassification distributions for the Septoria_leaf_spot classes for CCT and PVT, where both models managed to correctly classify 654 images out of 746 images. Meanwhile, the MaxViT architecture performs fairly well to classify this particular class. Overall, Septoria_leaf_spot is seemingly the class that requires more attention when the transformer models are trained.

#### 5.1.4. Area under Curve and Tabular Results

**Figure 12:**
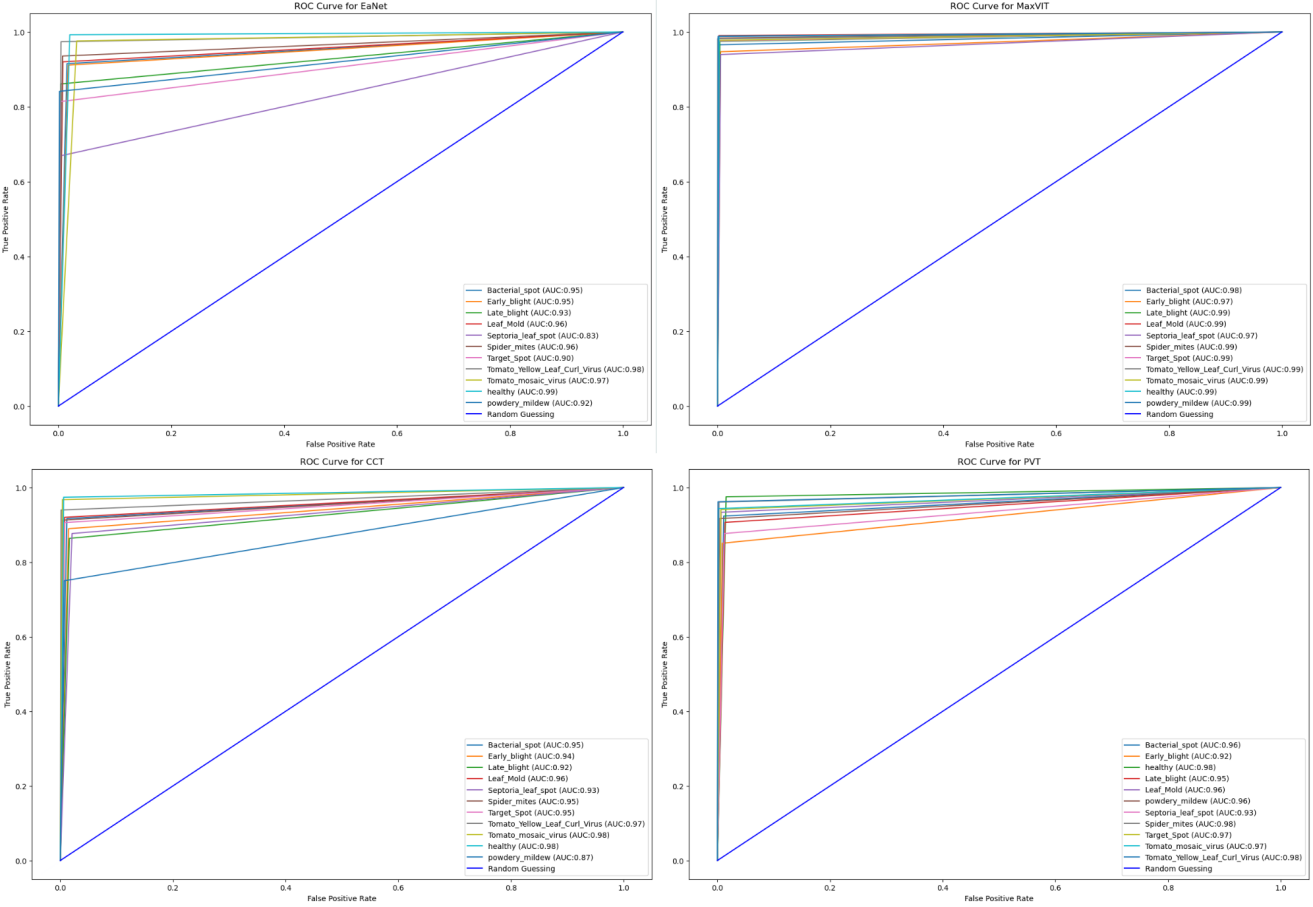
ROC for each of the models.

Finally, the Area Under Curve (AUC) for each model is showcased in Figure 12. Keeping consistency with high accuracy scores, MaxViT achieves the highest range of AUC scores between 97% to 99% for each individual class. PVT also showcases a consistent AUC score between 92% and 99%. EANet achieves the lowest AUC value of 83% for the Septoria_leaf_spot class while the lowest AUC value of CCT is for powdery mildew, with a score of 87%.

**Figure 13:**
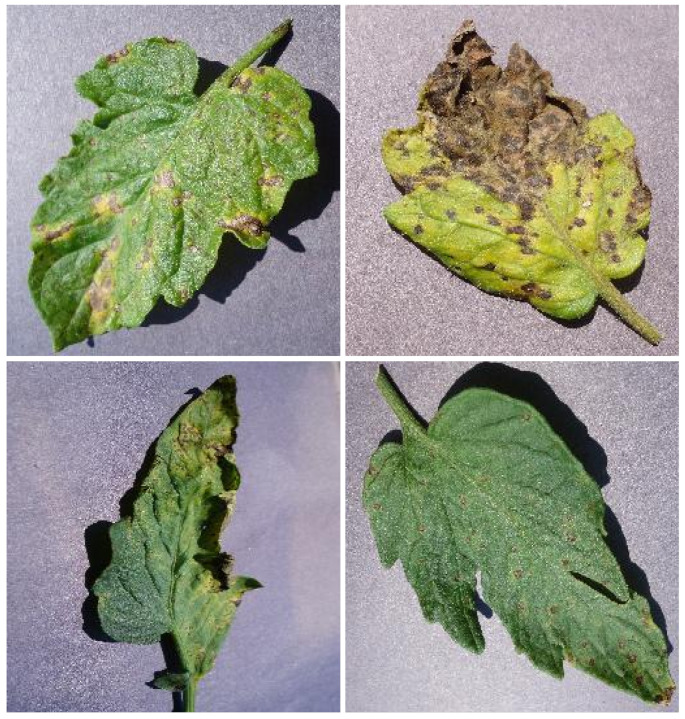
Inconsistent pattern in the Septoria Leaf Spot class.

A manual examination of the Septoria spot-affected leaves showcases an inconsistent pattern of spots ranging from slightly darkened areas to the whole leaf becoming yellow. There are also leaves with a sporadic pattern of darkened areas on them. A few examples of the pattern are showcased in Figure 13. To the best of our knowledge, perhaps this inconsistent pattern of spots is the reason for the smaller transformers not being able to properly classify it in many cases.

The generated results in Table 5 showcase that the MaxViT architecture comprehensively achieves better performance than the other models. The model achieves 97% accuracy over the dataset, with PVT being the distant second with 93% accuracy, CCT at third with 91%, and EANet at fourth with 89% accuracy. The PVT model employs a feature aggregation method that can handle images with different scales more effectively, this enables the model to produce better results. It is also noticeable that the accuracy of the models is relative to the number of total parameters. The MaxVit model having over 300 million parameters, nearly three times more than the next largest model PVT, achieves the highest score. Due to this sheer volume of parameters, the training time for MaxViT is also quite high. PVT, with the second largest number of parameters of more than 120 million, achieves the next best results. In terms of parameter size, CCT and EANet are the smallest two, resulting in the models achieving the lowest two scores, respectively. Due to having a large number of parameters, MaxViT and PVT also showcase smoother learning patterns with minimal symptoms of overfitting. Despite all the decent results, the MaxViT model obviously requires much more resources to be trained and deployed. Therefore, the speed of the model might create issues if it is to be implemented in a resource-constrained device, which should be a common scenario in a tomato cultivation field as expenditure savings play a crucial role here. In such cases, models such as EANet can be an excellent alternative as long as the users are okay with sacrificing some reliability.

Finally, the superior performance of the MaxViT architecture provides us with an understanding of the importance of combining local and global attention. There have been other architectures in the past that combine local and global attention information across a sub-portion of the network. However, MaxViT is the first architecture to combine local and global attention from a very early phase to the very last of the feature extraction layers. This combination of local+global attention throughout the entire network seems to have benefited tomato disease classification a lot, which is proven by the best result achieved by the network.

### 5.2. Results Generated on a Different Tomato Leaf Disease Dataset

#### 5.2.1. Accuracy and Loss Curves

To further verify our findings and to eliminate the possibility of having any biased conclusion based on just one dataset, we further checked for results on a smaller tomato leaf disease dataset. In the plantVillage dataset [30] we implemented the same four models and extracted results in terms of accuracy curves, loss curves, and classification reports. The setup of the models and other hyperparameters such as the number of epochs, learning rate, etc., has been kept the same as the run on the initial dataset.

The learning curves of the models on the second dataset further solidify our initial claims. Just like in the previous case, the EANet and the CCT models showcase much more fluctuated learning curves, showcasing hints of slight overfitting. The MaxViT and PVT models manage to achieve much smoother learning curves, representing the robustness of the learning for the models.

#### 5.2.2. Classification Report

**Figure 14:**
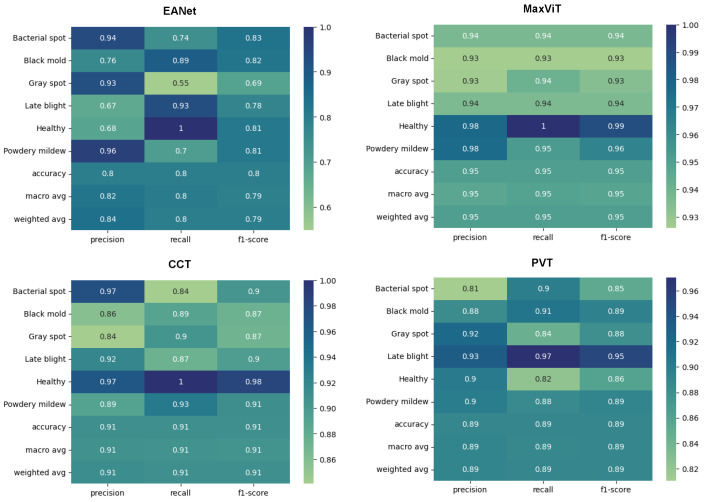
Classification report.

**Figure 15:**
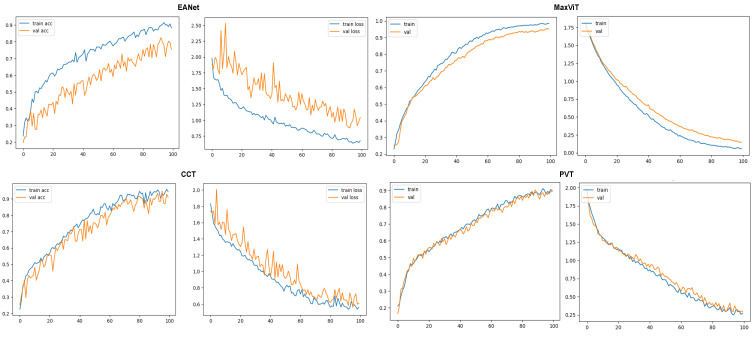
Accuracy and loss curves for the models.

The classification reports produced from the models also showcase in Figure 14 the superiority of MaxViT architecture to classify leaf diseases. MaxViT achieves 95% overall accuracy, much ahead of the next best produced by CCT, which is at 91%. The PVT and the EANet models fall in the 3rd and the 4th place with 89% and 80% overall accuracy, respectively. An important point to note here is that the results produced on [30] are not benchmark accuracy for these models on the respective dataset. As the second dataset has a much more complex background, the models are rather slow to train and the results would visibly improve if the training was run beyond the 100th epoch since the learning curve was still going up at the 100th epoch, which is visible in Figure 15. However, the target was to just keep the exact same experimental setup and to check the training pattern.

To sum up, after training on both datasets, we verified that MaxViT is indeed the superior architecture for tomato leaf disease classification. Therefore, MaxViT is the model to be deployed when tomato disease classification is needed. Meanwhile, if any low-resource model is needed for deployment, CCT and EANet can be decent alternatives.

## 6. Conclusions

To conclude, automated tomato leaf disease prediction can play an important role in taking preventive measures and improving the cultivation process. The most accessible method without the requirement for a professional is to photograph infected leaves and identify the disease type without any manual intervention. Therefore, four transformer-based models have been experimented with in the study for the classification of 11 different tomato leaf disease types from image data. Our empirical analysis showcases that the MaxViT architecture without any reservation is effectively the best transformer model to classify tomato leaf disease. The model beats all the other transformer models with a comprehensive 97% overall accuracy. Since the default MaxViT architecture has a huge number of parameters, future work may involve optimizing the default architecture to reduce the number of learnable weights with minimal sacrifice of accuracy. If it could be successfully carried out, the detection model should be easier to deploy on cheap embedded devices.

The models employed for this research work are mostly the default architectures of the existing model, with no specific modifications to account for the leaf images specifically that have been taken. Perhaps, it is possible to achieve even better scores by fine-tuning the existing models and by tweaking the architectures a little more. Additionally, we can experiment with the deployment of the model on edge devices. All these tasks will be part of future work.

## Figures and Tables

**Table 1:** Summary of related works on tomato leaf disease.

Paper	Dataset Name	Dataset Size	Algorithms	Accuracy	Comments
[7]	-	450	Alexnet	76.1%	Number of images are too small
			VGG16	55%	
			VGG19	55.6%	
[8]	PlantVillage [26]	13,262	AlexNet	97.49%	Variable image batch size is used which shows variations in the results for VGG16 but for AlexaNet shows limited variations.
			VGG16	97.29%	Only two deep learning methods are used for the analysis
[9]	PlantVillage [26]	14,828	AlexaNet	98.66%	Both deep learning and shallow models are used for comparison
			GoogleNet	99.18%	Deep learning models the better results
			SVM	94.54%	Both pre-trained and without pre-trained models are used
			Random Forest	95.47%	
[23]	PlantVillage [26]	18,160	Customized CNN	98.40%	A customize 8-layer CNN model has been used
					Manual augmentation technique was used
[25]	-	3000	CNN	98.49%	Dataset is not very large
					Only one method is used
[19]	PlantVillage [26]	-	DenseNet-77	99.98%	Total images used not mentioned
					Better mAP and test time
[27]	PlantVillage [26]	3900	DLQP+SVM	95.60%	Comparison with state of the art is missing
					Other classification tools can be used to check better classification accuracy
[28]	First hand data	1308	SLIC+SVM	98.50%	The proposed method can work having complex background
					Computation time is on the higher side

**Table 2:** Distribution of images among the classes in the first dataset.

Classes	Training	Validation
Bacterial Spot	2826	732
Early Blight	2455	643
Late Blight	3113	792
Leaf Mold	2754	739
Powdery Mildew	1004	252
Septoria Leaf Spot	2882	746
Spider Mites	1747	435
Target Spot	1827	457
Tomato Mosaic Virus	2153	584
Tomato Yellow Leaf	2039	498
Healthy	3051	806

**Table 3:** Distribution of images among the classes in the second dataset.

Classes	Training	Validation
Bacterial Spot	704	176
Black mold	428	108
Gray spot	537	135
Healthy	678	170
Late blight	627	157
Powdery mildew	1004	252

**Table 4:** Comparison of model parameters.

Models	Time Complexity (S)	Space Complexity (No. of Parameters)	Optimizer	Loss Function	Learning Rate	Weight Decay	Activation Function	Patch Size	Epochs
EANet [31]	2910	355,602	AdamW	Categorical Crossentropy	$1\times 10^{-3}$	0.0001	Softmax	2	100
MaxViT [32]	6531	30,429,907	AdamW	Categorical Crossentropy	$1\times 10^{-5}$	0	Softmax	-	100
CCT [33]	5839	408,268	AdamW	Categorical Crossentropy	$1\times 10^{-3}$	0.0001	Softmax	-	100
PVT [34]	6314	12,341,003	AdamW	Categorical Crossentropy	$5\times 10^{-5}$	0	Softmax	4	100

**Table 5:** Model Accuracies.

Models	Accuracy
MaxViT	97%
PVT	93%
CCT	91%
EANet	89%

## Data Availability

This dataset was collected from Kaggle, this can be found at https://www.kaggle.com/datasets/cookiefinder/tomato-disease-multiple-sources (accessed on 2 January 2023). This dataset was collected from Mendeley, this can be found at https://data.mendeley.com/datasets/ngdgg79rzb/1 (accessed on 17 March 2023).

## Abbreviations

The following abbreviations are used in this manuscript:

ViT	Vision Transformer
Deep Learning	DL
state-of-the-art	SOTA
CCT	Compact Convolutional Transformer
PVT	Pyramid Vision Transformer
MaxViT	Multi-Axis Vision Transformer
EANet	External Attention Network
